# Association between the traditional Chinese diet, non-communicable diseases and all-cause mortality: a longitudinal study based on China Health and Nutrition Survey (CHNS)

**DOI:** 10.1007/s00394-026-04057-w

**Published:** 2026-07-11

**Authors:** Jizhao Niu, Bai Li, Zumin Shi, Angeliki Papadaki

**Affiliations:** 1 Centre for Exercise, School for Policy Studies, Nutrition and Health Sciences, University of Bristol, 8 Priory Road, Bristol, BS8 1TZ UK; 2 Department of Nutrition Sciences, College of Health Sciences, QU Health, Qatar University, Doha, Qatar

**Keywords:** All-cause mortality, Dietary patterns, Non-communicable diseases, Traditional Chinese diet, Traditional diets

## Abstract

**Purpose:**

Traditional diets may reduce non-communicable disease (NCDs) risk. No research has examined associations between the traditional Chinese diet (TCD), defined using an a priori method, and NCD-related outcomes or all-cause mortality. This study aimed to assess the relationship between TCD adherence, NCD-related outcomes, and all-cause mortality.

**Methods:**

This longitudinal study utilised data from the China Health and Nutrition Survey (CHNS) between 1997 and 2011. Eligible participants were aged 18–74, had no NCDs at baseline, and participated in at least two survey waves. TCD adherence was assessed using a recently developed TCD index. Higher index scores indicate greater adherence; participants were categorised into low (0–6), medium (7–11), and high (12–23) adherence. Mixed-effects multilevel logistic regression and Cox proportional hazards models assessed associations with CVD, diabetes, cancer, obesity/central obesity, hypertension, and all-cause mortality.

**Results:**

Among 11,158 participants (mean age 42.2 years, 51.3% women), high TCD adherence was associated with lower risk of CVD (OR: 0.73, 95% CI 0.61, 0.87) and all-cause mortality (HR: 0.73, 95% CI 0.63, 0.87), but higher risk of obesity (OR: 1.19, 95% CI 1.08, 1.32) and central obesity (OR: 1.19, 95% CI 1.12, 1.26). However, when standardising the energy intake on a common basis, TCD adherence was inversely associated with obesity and central obesity.

**Conclusions:**

Higher adherence to the TCD was associated with reduced risk of CVD and all-cause mortality in this sample of Chinese adults. The positive association with obesity highlights the need to consider total energy intake in future dietary recommendations.

**Supplementary Information:**

The online version contains supplementary material available at 10.1007/s00394-026-04057-w.

## Background

Diet plays a crucial role in human health and wellbeing, and is recognised as a modifiable risk factor contributing to non-communicable disease (NCDs) and premature mortality [1]. According to the Global Burden of Disease study, deaths attributable to poor diet increased from 8 million in 1990 to 11 million in 2017 [2]. Cardiovascular diseases (CVDs) and type 2 diabetes were identified as the primary causes of diet-related mortality [3]. In 2021, poor diet accounted for 10.6% of all global deaths [4]. These findings highlight the potential of healthier dietary patterns to support longevity by promoting a healthy body weight, reducing inflammation, and improving metabolic function [5].

Traditional diets are long-established eating patterns that reflect a region’s culture and food environment [6]. These diets are commonly rich in plant-based and minimally processed foods, such as the Mediterranean and traditional Japanese diets, both of which are regarded as healthy diets and widely recommended for the prevention of NCDs [7, 8]. However, most existing evidence on traditional dietary patterns and health outcomes is based on dietary indices designed specifically for foods consumed among Western populations, such as the Mediterranean diet score [9], limiting their applicability to non-Western contexts.

In recent decades, dietary patterns in China have shifted from a traditional, plant-based diet to one characterised by increased consumption of refined grains and animal products [10]. This transition has been largely driven by agricultural development and rising income levels [10]. As a consequence, the health burden has grown substantially. In 2018, deaths related to NCDs accounted for approximately 88% of all deaths in the country [11]. Given the unique characteristics of the Chinese diet and the country’s growing burden of NCDs, it is essential to investigate how dietary patterns relate to health outcomes within this population [12].

A recent systematic review reported inconsistent findings regarding the association between the traditional Chinese diet (TCD) and health outcomes, largely due to variations in the definition of the TCD [13]. For instance, higher adherence to the TCD might be associated with lower risk of CVD [14], whereas the traditional Chinese northern diet might be positively associated with obesity compared with people following the traditional southern diet [15, 16]. Additionally, most studies in the review used a posteriori methods to define the TCD [17–19], which reflect current eating patterns but might not accurately capture the characteristics of the traditional diet [20]. This review also received numerous letters, highlighting the global research interest and the importance of advancing evidence on the health effects of the TCD [21].

To date, no study has examined the association between adherence to the TCD, health outcomes, and all-cause mortality in China, using an priori method to define this traditional diet [13]. A comprehensive index was recently developed, based on evidence from this recent systematic review [13] and the Delphi technique [22]. This index incorporates 15 food groups and one food-related habit that characterise the TCD, and can be used to investigate adherence to the TCD [22]. This study aims to apply this index, for the first time, to assess the relationship between adherence to the TCD, NCD-related outcomes, and all-cause mortality in the Chinese population.

## Methods

### Study design and participants

Dietary data for this study were obtained from the China Health and Nutrition Survey (CHNS) [23], a representative, continuous, open, prospective, household-based cohort study conducted in China [24]. Since 1989, ten waves of CHNS surveys have been conducted (1989, 1991, 1993, 1997, 2000, 2004, 2006, 2009, 2011 and 2015) using a multistage, stratified cluster random sampling, involving over 30,000 people from 7,200 households across 15 regions (initially nine provinces, with three cities added in 2011 and three provinces in 2015). These regions represent different levels of economic development, public resources, geographic characteristics, and population health, and so, as a whole, the survey is considered to be representative for the whole country [25]. Each survey wave collected data on demographics, physical activity (PA), dietary intake, and health status. Due to the availability of dietary data, this study utilised data collected from 1997 to 2011. Further details about the dataset and ethical approvals are provided in Supplementary Materials II (p. 1).

**Fig. 1:**
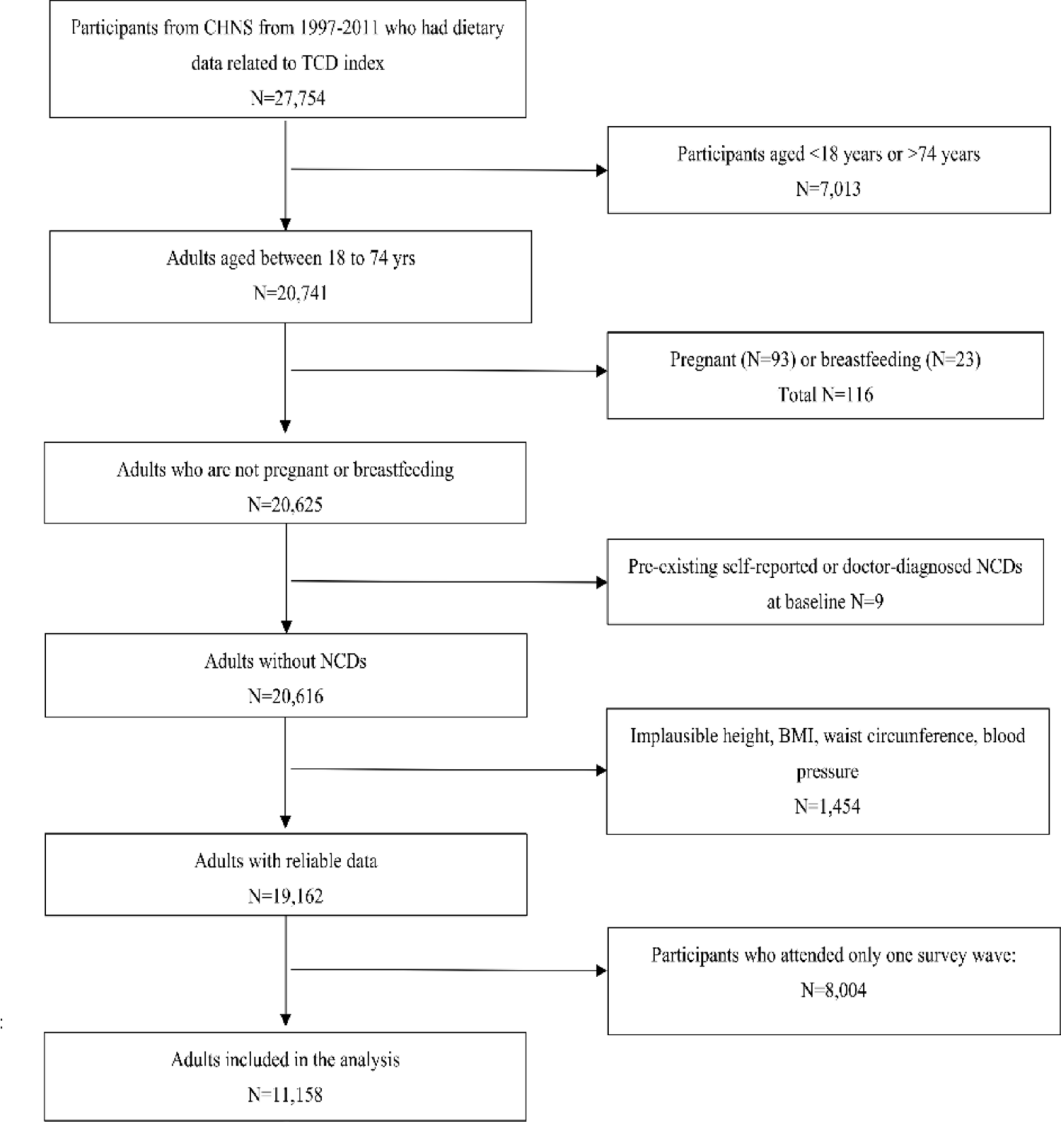
Flow chart of study participant selection. BMI, Body mass index; CHNS, Chinese Health Nutrition Survey; NCDs, Non-communicable diseases; TCD, Traditional Chinese diet

Between 1997 and 2011, a total of 27,754 participants provided dietary data related to the TCD index (Fig. 1). The following exclusion criteria were applied: individuals under the age of 18 or over 74; women who were pregnant or breastfeeding in a survey year; participants with self-reported or doctor-diagnosed NCDs at baseline; those with an outlying height (< 130 cm or > 200 cm), body mass index (BMI) (< 10 kg/m^2^ or > 58 kg/m^2^), waist circumference (WC) (< 50 cm or > 200 cm) or blood pressure (systolic blood pressure (< 80 mmHg) and diastolic blood pressure (< 50 mmHg) [26]; and those who participated in only one wave of the survey. After applying these criteria, 11,158 participants were included in the final analysis.

This study followed the Strengthening the Reporting of Observational Studies in Epidemiology (STROBE) reporting guideline (Supplementary Materials I) [27]. Details on ethical approval for the CHNS and this study are provided in Supplementary Materials II (p. 1).

### Dietary intake

Dietary intake data were collected by trained nutritionists on three consecutive days (two weekdays and one weekend day) at both the household and individual levels. Detailed information on dietary assessment is provided in Supplementary Materials II (p. 2). Food groups were coded using the Chinese Food Composition Tables (1991, 2002 and 2004 editions) [28–30], and the cumulative average intake for each food group was calculated (Supplementary Materials II, Table S1). For example, a participant’s intake of fruits was reported as *a*, *b* and *c* in the 1997, 2000 and 2004 CHNS waves, respectively. As such, ‘*a*’ represented the baseline intake; the cumulative average intake for 2000 would be calculated as (*a* + *b*)/2, and for 2004 as (*a* + *b* + *c*)/3. This method reflects participants’ dietary intakes over time and reduces random variability [31, 32].

### Calculation of the TCD adherence score

Adherence to the TCD was assessed using a newly developed a priori dietary index. This index was created based on evidence from a systematic review of the definition of the TCD [13] and established through a three-round Delphi study involving 58 nutrition experts, who reached consensus on its key components [22]. The resulting TCD index reflects core characteristics of the traditional Chinese dietary pattern, which includes 15 food groups and one eating habit, with scores ranging from 0 to 23 (Supplementary Materials II, Table S2). Each food group was assigned 1 or 2 points based on whether a participant’s intake met the recommended consumption criteria, which were informed by traditional dietary practices rather than modern nutritional guidelines. This index does not require consumption of all included food groups, but instead recognises the cumulative value of adherence to various elements of the traditional diet [22].

The TCD score was calculated for each participant at every survey wave, based on dietary intake data across all food groups. Higher scores indicate greater adherence to the TCD. Consistent with other widely used a priori dietary indices, such as the Mediterranean Diet Score [9] and the DASH diet index [33], we classified TCD adherence into three categories (low, medium, and high) using tertile-based cut-offs: low adherence (scores 0–6), medium adherence (scores 7–11) and high adherence (scores 12–23). This classification enables comparability across population subgroups and reflects a standard method for evaluating dietary adherence in nutritional epidemiology [34]. Additionally, the a priori nature of the TCD index lies in its expert-defined structure and scoring system, which were developed independently of the health outcomes assessed in this study and are designed to evaluate overall dietary patterns. This further supports its conceptual and methodological alignment with other traditional dietary indices used in international research [35].

### Outcome measures

NCD-related outcomes included CVD, diabetes, cancer, obesity, central obesity, and hypertension. CVD incidence was defined as self-reporting either having had a myocardial infarction or stroke, or currently using any CVD medications at any follow-up visits [36]; diabetes was defined based on a self-reported diagnosis, and/or having a fasting blood glucose ≥ 7.0 mmol/L or hemoglobinA1c (HbA1c) ≥ 6.5% (only in the 2009 wave), and/or or currently using antidiabetic drugs at any follow-up visit; cancer diagnoses were self-reported; obesity was defined as a BMI ≥ 28 kg/m^2^ according to the Working Group on Obesity in China [37]; central obesity was defined as having a waist circumference (WC ≥ 90 cm for men and ≥ 80 cm for women [38]; hypertension was defined as having an average systolic blood pressure (SBP) ≥ 140 mmHg or diastolic blood pressure ≥ 90 mmHg, or self-reporting a diagnosis of hypertension, or currently taking antihypertensive medication at any follow-up visits [39]. Anthropometric (height, body weight, WC) and blood pressure measurements were collected by trained health staff following standardised protocols recommended by the World Health Organisation [40, 41]. Detailed information concerning these outcome measures is provided in Supplementary Materials II, Table S3.

Data on all-cause mortality, including the reported date of death, were collected during household surveys conducted in each CHNS wave [42]. If multiple reports of death were recorded for the same participant, the earliest reported date was used for analysis. The baseline for each participant was defined as the date of their first participation in the study. Causes of death were not reported during the study period.

### Covariates

In each wave of the CHNS, data were collected using standardised and structured questionnaires, including participants’ self-reported age, sex, education level, marital status, annual household income (inflated to 2015 level), health status, smoking status, alcohol intake status, PA, personal medical history and measured anthropometric variables [24]. Potential confounding factors were identified based on the existing literature relevant to this study’s analysis, and included: age; sex; BMI; total energy intake, education level (low: primary school and lower; medium: junior middle school; high: college and higher); annual household income (low: CNY < 16,962; medium: CNY 16,962–39,590; high: CNY ≥ 39,590, based on tertiles of the distribution); residence location (urban or rural); PA level (very light, light, moderate, heavy and very heavy, according to participants’ self-reported activities, activity duration and work-related PA); smoking status (non-smokers, ex-smokers and current smokers, based on self-reported history); and alcohol consumption (yes or no, based on the question ‘Did you drink beer or any other alcoholic beverage during the last year?’); use of antihypertensive/antidiabetic medications (yes or no, based on self-reported information).

### Statistical analyses

Baseline characteristics of participants, categorised by TCD adherence level, were summarised using means and standard deviations (SD) for continuous variables or frequency and proportion (%) for categorical variables. Variations across TCD adherence categories were assessed using analysis of variance (ANOVA), the Kruskal–Wallis H test, or the Pearson’s Chi-squared test.

Total TCD scores, based on each food group’s cumulative average intake, were categorised into three tertiles: low (0–6), medium (7–11) and high (12–23) adherence. Associations between TCD adherence and categorical variables (CVD, diabetes, cancer, obesity, central obesity, and hypertension) were examined using mixed-effects multilevel logistic regression models, which incorporated time variations and random effects to account for the hierarchical structure of the data and unobserved heterogeneity. Three models were examined separately under adjusting energy and standardised frameworks: Model 1 (unadjusted); Model 2 (adjusted for age, sex, residence area, education level, annual household income, alcohol intake, smoking status, PA level, and energy intake); and Model 3 (adjusted for all variables in Model 2, in addition to BMI, hypertension medication and diabetes treatment). Since obesity is defined based on BMI values and central obesity is highly relevant to BMI, adjusting for BMI in logistic models may introduce multicollinearity [43]. This could lead to over-adjustment and potentially obscure the true influence of TCD adherence [44, 45]. Therefore, Model 3 was not used in the analyses of obesity or central obesity.

Cox proportional hazards models (with multivariable models adjusted similar to that described above) were used to assess the association between TCD adherence and all-cause mortality, with follow-up calculated from baseline until death, loss to follow-up, or the end of the 2011 survey (whichever occurred first). The hazard ratios (HRs) and 95% CIs were presented. The proportional hazard assumption was examined using the Schoenfeld residual test, with no violations detected [46].

Subgroup analyses were conducted to further examine the association between TCD adherence and all-cause mortality according to participant characteristics. Participants were stratified by key demographic characteristics and lifestyle factors, including age, sex, household income, education level, residence area, region, PA level, and smoking status. Within each subgroup, Cox proportional hazards models were used to estimate HRs and 95% CIs. Interaction effects between TCD adherence and each subgroup variable were assessed using the Wald test. Additionally, associations between each food group of the TCD index and all-cause mortality were assessed using standardised increments. The increment of each food group was calculated according to their SD (Supplementary Materials II, p. 7 and Table S4). Cox proportional hazards models were used to examine the influence of each food group on all-cause mortality.

Sensitivity analyses were conducted to assess the robustness of the main findings. First, to assess the independent effect of dietary structure and account for differences in total energy intake when assessing the associations between TCD adherence and health outcomes, the nutrient density method was used to standardise TCD scores [47]. Detailed information about this process is presented in Supplementary Materials II, p.7. This approach enabled comparisons of health outcomes across TCD adherence groups on a common energy basis, reflecting the quality and structure of dietary patterns at a fixed energy level [48]. Associations between TCD adherence and health outcomes were examined using the same multilevel logistic regression models as in the main analysis. Second, an alternative categorisation method was applied to TCD adherence, where participants were classified into three groups based on equal score distribution: low adherence (0–7), medium adherence (8–15), and high adherence (16–23). Third, instead of calculating scores based on the cumulative average intakes across each survey wave, the 3-day average intake of each food group was used. Fourth, to assess the validity of self-reported diabetes and the potential for under-ascertainment when relying on self-reported data alone, a concordance analysis was conducted among participants in the 2009 wave who had both self-report and biomarker data available. Biomarker-defined diabetes was defined as HbA1c ≥ 6.5% or fasting plasma glucose ≥ 7.0 mmol/L. Self-reported diabetes related information was defined as self-reported doctor-diagnosed diabetes or current use of antidiabetic medication, or current insulin use. This self-reported measure was compared with the biomarker definition, and Cohen’s κ, sensitivity, specificity, positive and negative predictive values, and overall agreement were calculated.

Stata/MP version 18.0 (StataCorp. 2021. Stata Statistical Software: Release 18. College Station, TX: StataCorp LLC) was used to conduct all statistical analyses. For all analyses, a two-sided *p* < 0.05 was considered statistically significant. The Bonferroni correction was applied to adjust the *p* values in each analysis [49]. The imputed data ranged at around 1% via multiple imputations.

## Results

Baseline participant characteristics are presented in Table 1. The participant selection process is shown in Fig. 1. After applying the exclusion criteria, a total of 11,158 participants were included in the analysis, with 51.3% females and 48.7% males. The mean age at baseline was 42.2 years (SD 13.3), and approximately two-thirds (65.6%) of participants resided in rural areas. Participants with high adherence to the TCD were more likely to be urban residents (40.7% vs. 32.7%), earn a higher annual household income (14.3% vs. 5.7%), and be non-smokers (72.2% vs. 65.4%), compared to those with low adherence.

**Table 1:** Baseline participant characteristics

Characteristics	Total participants	Adherence to the TCD (baseline by tertile), N (%)			*p* value^a^
		Low adherence (0–6)	Medium adherence (7–11)	High adherence (12–23)	
N	11,158	3327	4162	3669	
TCD score, mean (SD)	7.2 (4.6)	3.6 (2.0)	8.6 (1.3)	15.0 (2.3)	< 0.001
Age (year), mean (SD)	42.2 (13.3)	42.0 (13.4)	41.6 (13.2)	43.0 (13.0)	< 0.001
Sex					< 0.001
Men, n (%)	5431 (48.7%)	1727 (51.9%)	2101 (50.5%)	1603 (43.7%)	
Women, n (%)	5727 (51.3%)	1600 (48.1%)	2061 (49.5%)	2066 (56.3%)	
Education					< 0.001
Low, n (%)	8713 (78.1%)	2694 (81.0%)	3406 (81.8%)	2613 (71.2%)	
Medium, n (%)	2061 (18.5%)	552 (16.6%)	647 (15.5%)	862 (23.5%)	
High, n (%)	384 (3.4%)	81 (2.4%)	109 (2.6%)	194 (5.3%)	
Residence area					< 0.001
Rural, n (%)	7315 (65.6%)	2239 (67.3%)	2901 (69.7%)	2175 (59.3%)	
Urban, n (%)	3843 (34.4%)	1088 (32.7%)	1261 (30.3%)	1494 (40.7%)	
Household income per year (CNY/Y)^b^					< 0.001
Low, n (%)	6816 (61.1%)	2225 (66.9%)	2750 (66.1%)	1841 (50.2%)	
Medium, n (%)	3394 (30.4%)	913 (27.4%)	1179 (28.3%)	1302 (35.5%)	
High, n (%)	948 (8.5%)	189 (5.7%)	233 (5.6%)	526 (14.3%)	
Survey year					< 0.001
1997, n (%)	5375 (48.2%)	2017 (60.6%)	2335 (56.1%)	1023 (27.9%)	
2000, n (%)	2636 (23.6%)	813 (24.4%)	999 (24.0%)	824 (22.5%)	
2004, n (%)	1470 (13.2%)	257 (7.7%)	385 (9.3%)	828 (22.6%)	
2006, n (%)	755 (6.8%)	116 (3.5%)	225 (5.4%)	414 (11.3%)	
2009, n (%)	922 (8.3%)	124 (3.7%)	218 (5.2%)	580 (15.8%)	
BMI (kg/m^2^, mean (SD))	22.7 (3.2)	22.5 (3.1)	22.6 (3.1)	23.2 (3.3)	< 0.001
Smoking^c^					< 0.001
Non-smoker, n (%)	7568 (67.8%)	2176 (65.4%)	2744 (65.9%)	2648 (72.2%)	
Ex-smokers, n (%)	103 (0.9%)	17 (0.5%)	26 (0.6%)	60 (1.6%)	
Current smokers, n (%)	3487 (31.3%)	1134 (34.1%)	1392 (33.4%)	961 (26.2%)	
Alcohol drinking (yes, %)	4076 (36.5%)	1281 (38.5%)r	1557, 37.4%	1238, 33.7%	< 0.001
PA level^d^					< 0.001
No activity, n (%)	99, 0.9%	24, 0.7%	31, 0.7%	44, 1.2%	
Very light, n (%)	1956, 17.5%	431, 13.0%	587, 14.1%	938, 25.6%	
Light, n (%)	3170, 28.4%	911, 27.4%	1066, 25.6%	1193, 32.5%	
Moderate, n (%)	1655, 14.8%	486, 14.6%	597, 14.3%	572, 15.6%	
Heavy, n (%)	4211, 37.7%	1454, 43.7%	1846, 44.4%	911, 24.8%	
Very heavy, n (%)	67, 0.6%	21, 0.6%	35, 0.8%	11, 0.3%	

BMI, Body mass index; PA, Physical activity; SD, Standard deviation; TCD, Traditional Chinese diet

^a^Significance in chi-square test for categorical variables or ANOVA/Kruskal–Wallis for continuous variables

^b^CNY/Y = Chinese Yuan per year. Household income was calculated as the total income and revenue from all sources, subtracting expenditures, and then adjusted for inflation to reflect 2015 values in CNY/Yuan

^c^Based on self-reported history and current smoking status; smoking status was categorised into non-smokers, ex-smokers, and current smokers

^d^PA levels were categorised based on participants’ activities, activities’ duration and work-related PA

The average intakes of 15 food groups within the TCD index are shown in Supplementary Materials II, Table S5. The largest consumed food group was rice (272.5 g/day, SD 186.6), followed by wheat and wheat products (186.7 g/day, SD 169.7) and green leafy vegetables (171.1 g/day, SD 131.8). Compared to participants in the low adherence group, those in the high adherence group had a higher intake of rice (311.4 vs. 230.2 g/day, *p* < 0.001), green leafy vegetables (205.1 vs. 134.7 g/day, *p* < 0.001), corn and coarse grains (99.7 vs.67.2 g/day, *p* < 0.001) and non-green leafy green vegetables (161.9 vs. 107.9 g/day, *p* < 0.001), while participants with low adherence showed higher consumption levels of the other food groups (*p* < 0.001 for all). Additionally, the average intake for men was higher than that for women across all food groups, although the differences in wheat with filling and fruit consumption were not statistically significant.

### Associations between TCD adherence and NCD-related outcomes

The associations between TCD adherence and NCD-related outcomes are presented in Table 2. In Model 3, participants with a high TCD adherence had a 27% lower risk of CVD incidence (OR: 0.73, 95% CI 0.61, 0.87). Additionally, inverse associations between high, compared to low adherence to the TCD, were observed for diabetes (OR: 0.74, 95% CI 0.56, 0.98) and hypertension (OR: 0.88, 95% CI 0.79, 0.97), but only in Model 2. No association was found between TCD adherence and cancer incidence.

**Table 2:** Associations between TCD adherence and NCD-related outcomes, CHNS, 1997–2011*

NCD-related outcomes	Adherence to the TCD					*p* for trend***
	Low adherence (0–6)	Medium adherence (7–11)	*p* value**	High adherence (12–23)	*p* value**	
	OR (95% CI)	OR (95% CI)		OR (95% CI)		
CVD						
Model 1	Reference	0.70 (0.61, 0.81)	< 0.001	0.55 (0.46, 0.65)	< 0.001	< 0.001
Model 2	Reference	0.79 (0.68, 0.92)	0.002	0.69 (0.58, 0.83)	< 0.001	< 0.001
Model 3	Reference	0.79 (0.68, 0.92)	0.002	0.73 (0.61, 0.87)	0.001	< 0.001
Diabetes						
Model 1	Reference	0.77 (0.66, 0.89)	0.001	0.61 (0.51, 0.73)	< 0.001	0.004
Model 2	Reference	1.04 (0.84, 1.29)	0.704	0.74 (0.56, 0.98)	0.036	0.085
Model 3	Reference	1.24 (0.96, 1.62)	0.103	0.85 (0.61, 1.19)	0.350	0.650
Cancer						
Model 1	Reference	1.09 (0.67, 1.75)	0.732	0.76 (0.40, 1.45)	0.414	0.538
Model 2	Reference	1.15 (0.71, 1.86)	0.570	0.88 (0.46, 1.68)	0.688	0.859
Model 3	Reference	1.15 (0.71, 1.86)	0.523	0.88 (0.46, 1.67)	0.682	0.852
Obesity						
Model 1	Reference	1.15 (1.04, 1.26)	0.004	1.32 (1.20, 1.45)	< 0.001	< 0.001
Model 2	Reference	1.10 (1.00, 1.21)	0.042	1.19 (1.08, 1.32)	0.001	0.001
Model 3	Reference	NA	NA	NA	NA	NA
Central obesity						
Model 1	Reference	1.12 (1.06, 1.18)	< 0.001	1.38 (1.30, 1.46)	< 0.001	< 0.001
Model 2	Reference	1.05 (0.99, 1.11)	0.064	1.19 (1.12, 1.26)	< 0.001	< 0.001
Model 3	Reference	NA	NA	NA	NA	NA
Hypertension						
Model 1	Reference	0.86 (0.79, 0.94)	0.001	0.73 (0.66, 0.80)	< 0.001	< 0.001
Model 2	Reference	0.95 (0.87, 1.04)	0.290	0.88 (0.79, 0.97)	0.012	0.013
Model 3	Reference	0.90 (0.77, 1.05)	0.188	0.89 (0.75, 1.05)	0.178	0.167

Model 1: Crude model

Model 2: Adjusted for age, sex, residence area (urban/rural), education level, household annual income, alcohol consumption (yes/no), smoking status, PA level and energy intake

Model 3: Adjusted for variables in Model 2, in addition to BMI, anti-hypertension medication and diabetes treatment

All adjusted variables were treated as time-varying covariates

CHNS, Chinese Health Nutrition Survey; CIs, Confidence intervals; NCD, Non-communicable disease; OR, Odds ratio; TCD, Traditional Chinese diet; NA, Not applicable

*All analyses were conducted using multilevel logistic regression models incorporating time variations and random effects

**The *p*-value was adjusted (*p* < 0.008) for multiple comparisons using the Bonferroni correction

***This *p*-value was used to assess the linear trend

In contrast, in Model 2, high TCD adherence was associated with increased risk of obesity (OR: 1.19, 95% CI 1.08–1.32) and central obesity (OR: 1.19, 95% CI 1.12–1.26). However, these associations were reversed when using an energy-standardised approach based on the nutrient density method, which reflects dietary structure under a fixed energy intake (2000 kcal/day for women and 2500 kcal/day for men). Under this approach, high TCD adherence was associated with lower risk of obesity (OR: 0.86, 95% CI 0.78–0.95) and central obesity (OR: 0.85, 95% CI 0.80–0.91). These findings are further explored in the “Discussion” section.

### Associations between TCD adherence and all-cause mortality

After a mean follow-up of 8.6 years (median, 9 years), among a total of 95,932 person-years of follow-up, 586 deaths were observed. In Model 3, compared to the low TCD adherence group, participants with high adherence to the TCD had a 27% lower risk of all-cause mortality (HR, 0.73, 95% CI 0.63, 0.87) (Table 3). When adherence to the TCD was assessed continuously, each standard deviation (SD) score increment was associated with a 7% decreased risk of all-cause mortality (HR, 0.93, 95% CI 0.90, 0.97).

**Table 3:** Associations between TCD adherence and all-cause mortality, CHNS, 1997–2011*

Variable	Adherence to the TCD						
	Low adherence (0–6)	Medium adherence (7–11)	*p* value	High adherence (12–23)	*p* value	Per SD TCD score increment	*p* value
Person-year of follow-up	19,900	33,313		42,392		95,605	
Events, No	189	226		171		586	
Model 1 (HR, 95% CI)	Reference	0.70 (0.57,0.84)	< 0.001	0.61 (0.53,0.70)	< 0.001	0.72 (0.64, 0.80)	< 0.001
Model 2 (HR, 95% CI)	Reference	0.78 (0.64,0.95)	0.013	0.73 (0.62,0.86)	< 0.001	0.93 (0.89, 0.96)	< 0.001
Model 3 (HR, 95% CI)	Reference	0.78 (0.64,0.95)	0.013	0.73 (0.63,0.87)	< 0.001	0.93 (0.90, 0.97)	< 0.001

Model 1: Crude model

Model 2: Adjusted for age, sex, residence area (urban/rural), education level, household annual income, alcohol consumption (yes/no), smoking status, PA level, and energy intake

Model 3: Adjusted for variables in Model 2, in addition to BMI, anti-hypertension medication and diabetes treatment

All adjusted variables were treated as time-varying covariates

CHNS, Chinese Health Nutrition Survey; CIs, Confidence intervals; HR, Hazard ratio; SD, Standard deviation; TCD, Traditional Chinese diet

*Analyses were conducted using Cox proportional hazards models

### Associations between TCD adherence and all-cause mortality by presence of risk factors

Subgroup analyses for the adherence to the TCD and all-cause mortality, according to key demographic characteristics and lifestyle factors, are presented in Supplementary Materials II, Table S6. A protective association of high adherence to the TCD was observed across most subgroups, with stronger effects in those aged between 35 and 64 (HR, 0.60, 95% CI 0.47, 0.77), men (HR, 0.58, 95% CI 0.47, 0.73), low-income groups (HR, 0.45, 95% CI 0.34, 0.61), low-education groups (HR, 0.53, 95% CI 0.43, 0.67), those living in rural areas (HR, 0.54, 95% CI 0.42, 0.69) and in southern China (HR, 0.53, 95% CI 0.40, 0.69), having very light PA levels (HR, 0.47, 95% CI 0.33, 0.69) and non-smokers (HR, 0.45, 95% CI 0.34, 0.59).

### Associations between food groups and all-cause mortality

**Fig. 2:**
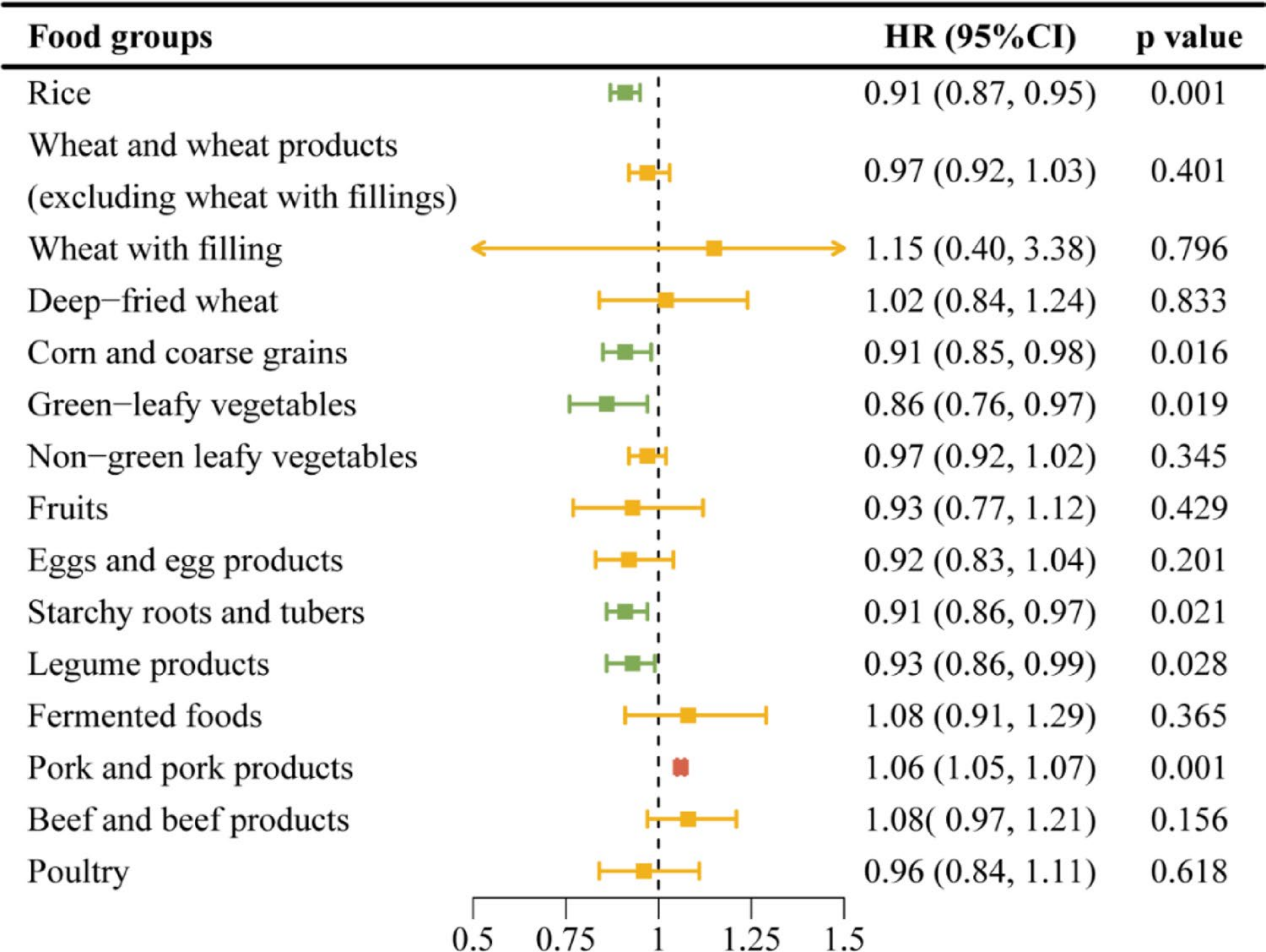
Associations between food groups included in the TCD index and all-cause mortality, CHNS, 1997–2011. CHNS, Chinese Health Nutrition Survey; CIs, Confidence intervals; HR, Hazard ratio; TCD, Traditional Chinese diet

Explorations of the associations between each food group of the TCD index and all-cause mortality are shown in Fig. 2. Higher consumption of rice (HR, 0.91, 95% CI 0.87, 0.95), corn and coarse grains (HR, 0.91, 95% CI 0.85, 0.98), leafy green vegetables (HR, 0.86, 95% CI 0.76, 0.97), starchy roots and tubers (HR, 0.91, 95% CI 0.86, 0.97), and legume products (HR, 0.93, 95% CI 0.86, 0.99) was associated with lower all-cause mortality, while the consumption of pork and pork products was related to higher all-cause mortality (HR, 1.06, 95% CI 1.05, 1.07). There were no significant associations between the other food groups and all-cause mortality.

### Sensitive analyses

All sensitivity analyses are presented in Supplementary Materials II (p.13–p.18). First, using the nutrient density method to standardise energy intake (Supplementary Materials II, Table S7a, b) yielded consistent inverse associations between TCD adherence and CVD, hypertension, and all-cause mortality. Notably, the previously observed positive associations with obesity and central obesity were reversed under the energy-standardised framework. Specifically, high TCD adherence was associated with lower risks of obesity (OR: 0.86, 95% CI 0.78–0.95) and central obesity (OR: 0.85, 95% CI 0.80–0.91), suggesting that these associations may be driven by differences in total energy intake rather than dietary structure alone.

Second, results remained largely consistent when an alternative classification approach was applied, categorising participants into low (0–7), medium (8–15), and high (16–23) adherence groups based on equal score distribution (Supplementary Materials II, Table S7c, d). Although the association with diabetes was no longer statistically significant, the direction of the effect remained similar, likely reflecting differences in sample distribution and statistical power.

Third, when calculating the TCD score using the 3-day average intake instead of cumulative means, slight changes were observed in total follow-up years and death counts, due to fluctuations in participant inclusion. However, the overall associations between TCD adherence and both NCD outcomes and all-cause mortality remained stable (Supplementary Materials II, Table S7e, f).

In the 2009 subgroup with available HbA1c and fasting blood glucose data, self-reported diabetes showed fair agreement with blood-based diabetes indicators, with a Cohen’s κ of 0.31 (95% CI 98.8–99.3%), sensitivity of 22.2% (95% CI 19.0–25.8%), specificity of 99.1%, and overall agreement of 91.3% (Supplementary Materials II, Table S9). This indicates that self-reported diabetes had very high specificity but low sensitivity when compared with blood-based indicators, suggesting that relying on self-report alone would miss a substantial proportion of diabetes cases identified through biochemical measures. This confirms that the composite definition adopted in the present analysis is unlikely to be inflated by self-report false positives.

## Discussion

To our knowledge, this is the first study to use an a priori method to assess adherence to the TCD and examine the association between adherence to this traditional dietary pattern, NCD-related outcomes and all-cause mortality. By utilising a comprehensive, evidence-based index of TCD adherence [22], this study addresses limitations of earlier research examining these associations using a posteriori indices [13], as the latter often reflect current dietary practices rather than characteristics of the traditional diet.

In this representative sample of Chinese adults, high, compared to low, adherence to the TCD was associated with a 27% lower risk of CVD, a 27% decreased risk in all-cause mortality, and a 19% higher risk of obesity and central obesity. However, the latter association should be interpreted with caution due to the potential influence of total energy intake.

### Association between TCD adherence and NCD-related outcomes

The paradoxical finding that high TCD adherence is associated with a reduced risk of CVD but a higher risk of obesity and central obesity, underscores the complex relationship between dietary quality, energy intake, and NCD risk. The observed inverse association between TCD adherence and CVD risk is consistent with findings from previous studies [17, 50, 51]. Compared to the low adherence group, participants with high TCD adherence consumed more rice, green leafy vegetables and non-green leafy vegetables, while having lower intakes of meat. This type of eating pattern has been associated with lower SBP and improved lipid profiles, which could protect against CVD [50]. When interpreting the association between TCD adherence and NCD-related outcomes, it is important to consider the cumulative effect of the overall dietary pattern rather than individual foods. For instance, fruits have been reported to significantly decrease the risk of CVD [52]. Although the high adherence group had a lower intake of fruits compared to the low adherence group, it consumed more rice and green leafy vegetables, and fewer wheat products. The overall combination of these food groups might therefore have contributed to the favourable CVD outcomes observed [53, 54].

Although TCD is rich in plant-based foods, higher adherence may also involve increased consumption of traditional staples such as refined rice and pork, potentially leading to higher energy intake and subsequent weight gain. This is particularly relevant in populations that have undergone rapid nutrition and economic transitions, where nutrient-rich diets may coexist with increased obesity prevalence [55, 56]. Additionally, in China, vegetables are typically stir-fried rather than consumed raw, resulting in additional caloric intake from cooking oils [57]. Although the TCD index included one cooking-related habit, namely cooking by steaming and/or boiling, this component only partly captured food preparation practices, as it did not quantify the amount of cooking oil used or identify the preparation method of each specific food group. Therefore, additional energy intake from oil-based cooking methods, particularly stir-frying of vegetables, may not have been fully accounted for. In this study, participants in the high adherence group consumed markedly higher quantities of vegetables, which could have contributed to increased energy intake and therefore to elevated risk of obesity and central obesity. However, when energy intake was standardised using the nutrient density method, the association between TCD adherence and obesity outcomes was reversed. Under this approach, higher TCD adherence was associated with reduced risks of both obesity and central obesity. This finding suggests that the positive association observed in the primary analysis may be attributable to greater total energy intake rather than the TCD pattern itself. These results underscore the importance of considering energy intake when assessing dietary quality and support the use of energy-adjusted methods in dietary epidemiology [58]. Future studies should consider refining the TCD index to better control for differences in total energy intake, allowing for a clearer interpretation of the association between traditional dietary patterns and health outcomes.

The associations between TCD adherence, hypertension and diabetes showed a protective trend, although they were not statistically significant in the fully adjusted models. The TCD index includes various food groups in suggested quantities which are aligned with Chinese Dietary Guidelines, as illustrated in the Chinese Food Pagoda [59]. These food groups, consumed in the recommended quantities, provide essential vitamins, minerals and fibre that have anti-inflammatory and antioxidant properties, which may reduce the risk of type 2 diabetes and all-cause mortality [47, 60]. In partially adjusted models, which excluded BMI, antihypertensive medication, and diabetes treatment, significant inverse associations were observed. However, in the fully adjusted model, high adherence to the TCD was not significantly associated with hypertension or diabetes. These differences may reflect the mediating role of these variables in the causal pathway [61]. Future research should incorporate formal mediation analyses to clarify the direct and indirect pathways through which TCD adherence influences these outcomes.

In addition, the non-significant association with diabetes should be interpreted in light of diabetes ascertainment. In the 2009 subgroup with available blood-based biomarkers, self-reported diabetes showed fair agreement with blood-based diabetes indicators, with very high specificity but low sensitivity. This suggests that participants who self-reported having diabetes were likely to be true cases, but that many participants meeting biochemical criteria for diabetes did not report having been diagnosed. Therefore, relying on self-reported diagnosis alone may lead to under-ascertainment of diabetes, particularly in survey waves without HbA1c data, and may have contributed to the non-significant association observed for diabetes. This finding is consistent with previous research among middle-aged and older Chinese adults, which showed that self-reported diabetes has high specificity but limited sensitivity [62]. Evidence also suggests that incorporating HbA1c can identify additional diabetes cases that would otherwise be missed [62–64], and that diabetes reporting may be poorer among rural residents than urban residents, which is relevant given the socioeconomic and geographic differences in both diabetes awareness and dietary patterns [62]. Future studies should incorporate repeated biomarker measurements, including HbA1c where possible, to improve diabetes ascertainment and reduce potential under-reporting.

No significant association was observed between TCD adherence and cancer, which may be due to the limited number of cancer cases in the current sample, potentially reducing statistical power. Additionally, cancer development is influenced by long latency periods, multifactorial aetiology, and potential gene-diet interactions that were not assessed in this study [65, 66]. Future longitudinal studies with extended follow-up durations and larger sample sizes of incident cancer cases are needed to clarify whether the TCD confers protective or adverse effects for this outcome.

### TCD adherence and all-cause mortality

Few studies have examined the association between TCD adherence and all-cause mortality. This study demonstrated that higher adherence to the TCD is associated with a lower risk of all-cause mortality. This finding aligns with previous evidence highlighting the protective effects of fruit- and vegetable-rich diets, particularly in Chinese populations [67]. Subgroup analyses further demonstrated consistent protective associations between high adherence to the TCD and all-cause mortality, particularly among socioeconomically disadvantaged groups, such as those with low income, low education levels and living in rural areas, which might be due to their higher baseline risk. A previous systematic review indicated that individuals with low socioeconomic status (SES) are more likely to consume unhealthy diets [68]. Consistent with this, baseline data showed that participants with low-income, low-education and living in rural areas had substantially lower mean TCD scores than their counterparts, and a majority of low-income, low-education and rural participants fell in the low-adherence group at baseline (Supplementary Materials II, Table S8). Within these groups, therefore, moving into the high-adherence tertile reflects a larger ‘absolute improvement’ in overall dietary quality than the same shift would represent in higher-SES groups, providing a plausible mechanism for the larger HR reductions observed, which is consistent with evidence that dietary-intervention benefits are typically largest in populations whose baseline diets are of lower quality.

As such, the promotion of the TCD, which contains diverse food groups, alongside culturally relevant and appropriate consumption guidelines, could be a cost-effective strategy to reduce NCD burden and all-cause mortality [17, 50]. Future investigations should assess the potential for promoting the TCD as a targeted intervention strategy in vulnerable populations.

### Individual food groups and all-cause mortality

This study also assessed the association between individual food groups that characterise the TCD and all-cause mortality by using standardised incremental models, with findings that are partly consistent with previous studies. Previous studies have reported inverse associations between the consumption of rice, whole grains, green leafy vegetables and starchy foods and the risk of all-cause mortality [69–71]. The current study showed similar results for these food groups. However, no consistent results have been found for the association between pork and all-cause mortality. While processed red meat and pork have been linked to an increased risk of all-cause mortality and cancer [72], a systematic review conducted among Asian populations reported an inverse association between meat and mortality risk [73]. These discrepancies may reflect broader contextual factors, such as the rising prevalence of NCD risk factors in Asian countries. In these settings, meat consumption may contribute less to mortality compared to other dominant risk factors, including low SES, physical inactivity, and obesity or adiposity [73]. Additionally, smoking still remains a leading contributor to cancer incidence in Asia and may confound associations with dietary factors [74]. Given these complexities, future studies should disentangle the contributions of dietary patterns, and individual food groups within those, from other lifestyle and sociodemographic factors, to identify which contribute the most. Additional research is also needed to clarify the health effects of traditional animal-based foods such as pork, considering preparation methods (e.g., preservation, cooking style), within the broader context of the overall dietary pattern.

### Strengths and limitations

To the best of our knowledge, this is the first prospective longitudinal study in China to use an a priori method to assess the association between TCD adherence, NCD-related outcomes and all-cause mortality, thereby addressing limitations of a posteriori methods in such analyses [20]. The strengths of this study include a large sample size from a representative survey, a long-term follow-up period and detailed information on confounding variables. The use of mixed-effects multilevel logistic regression models, incorporating time variations and random effects, allows for more accurate estimation of effects over time. To reduce the risk of reverse causation, individuals with pre-existing NCDs were excluded. Additionally, the use of cumulative average intakes enhanced the accuracy of estimating long-term dietary intake. Furthermore, the application of the nutrient density method enabled a robust assessment of the association between TCD adherence and health outcomes, independent of energy intake. Moreover, multiple sensitivity analyses were conducted to ensure the robustness and reliability of the findings.

However, several limitations should be acknowledged. First, most outcomes were self-reported and not validated by medical records, which might contribute to over- or underestimation of events. Recall bias may have occurred if participants forgot previous diagnoses or reported them inaccurately during long-term follow up. Reporting bias may also have occurred because disease reporting may differ by factors such as health awareness, socio-economic position, area of residence, and access to healthcare. As these factors may also be related to TCD adherence, differential outcome misclassification cannot be fully excluded. The CHNS dataset did not include medical record verified outcome events, so we were unable to directly quantify the impact of this bias on the estimated ORs. Published validation studies in Chinese cohorts suggest that self-reported CVD and diabetes generally have high specificity but more limited sensitivity, meaning that true incident cases are more likely to be under-ascertained than over-ascertained [62, 75, 76]. Therefore, some of the observed associations may be conservative. However, protective associations were also observed for all-cause mortality, which is less susceptible to individual concealment, and findings were generally robust across multiple sensitivity analyses. In addition, the positive association observed for objectively measured obesity and central obesity is not consistent with a general pattern of systematic concealment of adverse outcomes among participants with high TCD adherence. Therefore, reporting bias is unlikely to fully explain the overall pattern of findings. For example, for diabetes, case ascertainment was strengthened by incorporating a blood biomarker definition in the 2009 wave and self-reporting antidiabetic medication use in any follow-up visits. However, because HbA1c was only available in 2009 and antidiabetic medication use was also self-reported, some diabetes cases may still have been missed, although the use of a composite diabetes definition in this study has been supported.

Second, although extensive adjustments were conducted for covariates, residual confounding from unmeasured or unrecorded risk factors (for example lifestyle factors like stress and sleep), cannot be entirely ruled out. Third, this study used data from 1997 to 2011, which might raise concerns about relevance to current dietary practices. However, the purpose of this study was to evaluate whether higher adherence to a predefined traditional Chinese dietary pattern was associated with subsequent health outcomes, rather than to describe current Chinese dietary habits. The 1997 to 2011 CHNS data are appropriate for this purpose because traditional dietary characteristics were still more observable during this period, allowing sufficient variation in TCD adherence to assess its associations with NCD-related outcomes and all-cause mortality. Evidence from later CHNS waves and more recent surveillance data suggests that China’s dietary transition has continued after 2011 [77], with shifts toward lower carbohydrate intake [78, 79], higher fat intake [78, 79], and greater consumption of energy-dense [78, 79] and ultra-processed foods [80, 81]. These trends indicate that the traditional dietary pattern examined in this study is being progressively displaced. This, in turn, highlights the relevance of examining its potential long-term health implications, rather than as a description of current dietary habits in China. Nevertheless, the magnitude of the associations observed in this study should be extrapolated to post-2020 Chinese populations with caution, because contemporary dietary behaviours, food environments and total energy intake may differ from those observed during the study period. Future studies should validate, and potentially update, the TCD index using more recent data.

In addition, although the TCD index included one cooking-related habit, this item only partly captured food preparation practices. It did not quantify the amount of cooking oil used or identify the preparation method of each specific food group. Therefore, additional energy intake from oil-based cooking methods may not have been fully captured. Future studies should collect more detailed information on food group specific preparation methods and individual level cooking oil consumption, to allow a more comprehensive assessment of dietary quality.

## Conclusion

Adherence to the TCD may be beneficial for reducing the risk of CVD and all-cause mortality. However, it may also be associated with an increased risk of obesity and central obesity, potentially due to higher energy intake among individuals with greater adherence. When standardising energy intake, adherence to the TCD appears to show a protective association with obesity and central obesity, suggesting that total energy intake influences these associations. Future studies should refine the TCD index used in this study to better control for energy intake, while maximising its health benefits. Potential directions for refinement include introducing both lower and upper intake thresholds for energy-dense traditional components, such as refined grains and pork, and incorporating components that reflect contemporary dietary risks, including added sugar, sugary beverages, sodium, fried foods and ultra-processed foods. These refinements would help distinguish the healthful structure of the TCD from excessive total energy intake in modern dietary contexts. Additional research is also needed to explore the potential of the TCD as a targeted intervention strategy to improve public health, particularly in vulnerable populations. Moreover, applying this TCD index to Chinese populations living outside China would enable cross-country comparisons, including among first- and second-generation Chinese migrants worldwide.

## Supplementary Information

Below is the link to the electronic supplementary material.

Supplementary Material 1

Supplementary Material 2

## Data Availability

The China Health and Nutrition Survey data are available online (https://www.cpc.unc.edu/projects/china).
