# Supplementary material for: Association between the traditional Chinese diet, non-communicable diseases and all-cause mortality: a longitudinal study based on China Health and Nutrition Survey (CHNS)
Source: Eur J Nutr. 2026 Jul 11;65(5):209. doi: 10.1007/s00394-026-04057-w (PMC13356078; doi:10.1007/s00394-026-04057-w)
Supplement: Supplementary file 2 — Supplementary Material 2 [file 394_2026_4057_MOESM2_ESM.docx]

**Supplementary material II**

**1. Description of dataset and ethical approval**

The China Health and Nutrition Survey (CHNS) is a nationally representative, long-term follow-up survey initiated in 1989. Over the years, the CHNS has conducted 10 rounds of data collection, in 1989, 1991, 1993, 1997, 2000, 2004, 2006, 2009, 2011, and 2015. The survey has covered 12 provinces and three megacities in China, with three cities added in 2011 and three provinces in 2015. These regions represent different levels of economic development, public resources, geographic characteristics, and population health. To generate a representative sample size, a multistage, random-cluster sampling method was used for participant selection in each region. The study’s protocols, instruments, and informed consent process received ethical approval from the Institutional Review Boards of both University of North Carolina Chapel Hill and the Chinese CDC, with all participants providing written informed consent (<http://www.cpc.unc.edu/projects/china/data/data.html>). However, data for 2015 were not available online. Additionally, the food codes for nutrition data were not accessible for waves 1989 to 1993. As such, this study used data from 1997 to 2011.

For this study, all procedures were conducted in accordance with the guidelines outlined in the Declaration of Helsinki. The study received approval from the School for Policy Studies Research Ethics Committee at the University of Bristol.

**References**

1. China Health and Nutrition Survey — China Health and Nutrition Survey (CHNS)

[Internet]. Available from: https://www.cpc.unc.edu/projects/china

2. Zhang B, Zhai FY, Du SF, Popkin BM. The C hina Health and Nutrition Survey, 1989–

2011. Obesity Reviews 2014;15:2–7.

3. Popkin BM, Du S, Zhai F, Zhang B. Cohort Profile: The China Health and Nutrition

Survey—monitoring and understanding socio-economic and health change in China, 1989–2011.International journal of epidemiology Oxford University Press; 2010;39:1435–40.

**2. Description of dietary intake assessment**

The dietary assessment method has been detailed in previous publications (2). In brief, individual dietary data were collected through face-to-face interviews during the baseline survey. These assessments involved gathering information on participants' dietary habits over three separate 24-hour periods. Participants were asked to report all foods and beverages consumed within a 24-hour timeframe. The types and quantities of food items were recorded using visual aids and standardised measurements, including household containers and packaging labels indicating weight in grams. All collected data were measured by cooked weight. To ensure representative data, dietary assessments were conducted over three randomly selected consecutive days, including two weekdays and one weekend day, balancing sampling across all seven days of the week.

The CHNS dietary records included 2,243 food items, categorised using the Chinese Food Composition Table (FCT). Different survey waves used different versions of the FCT, and the version corresponding to this study is presented in Table S1. Specifically, the 1991 FCT was used to estimate nutrient values for dietary data from 2000 and earlier. This version marked a significant improvement over previous editions due to higher-quality chemical analyses and enhanced methodologies for deriving average nutrient values, accounting for geographic variations in food composition across the country. In collaboration with the National Institute of Nutrition and Food Safety, the University of North Carolina Chapel Hill and Chinese CDC team contributed to updating and refining this FCT. For the 2004 survey, the 2002 FCT was applied, while the most recent version at the time (2004) was used for the 2006 survey.

The cumulative average intake of each food group was used in this study. Each individual's intake during each survey wave was calculated to determine the cumulative average. Specifically, the 1997 intake served as the baseline for the follow-up period between 1997 and 2000. The average intake from 1997, 2000, and 2004 was used for the follow-up between 2000 and 2004. For example, if a person’s fruit intake was a, b, and c in 1997, 2000, and 2004, respectively, ‘a’ represents the baseline intake. The cumulative average fruit intake would be ‘a’ in 1997, (a + b)/2 in 2000, and (a + b + c)/3 in 2004. The dietary intake data used for calculating cumulative averages is presented in Table S1.

**References**

1. Zhang B, Zhai FY, Du SF, Popkin BM. The China Health and Nutrition Survey, 1989–

2011. Obesity Reviews 2014;15:2–7.

2. Popkin BM, Du S, Zhai F, Zhang B. Cohort Profile: The China Health and Nutrition

Survey—monitoring and understanding socio-economic and health change in China, 1989–2011.International journal of epidemiology Oxford University Press; 2010;39:1435–40.

**3.** **Table S1. Chinese Food Composition Table version used and data used to calculate cumulative average intakes**

| **Wave** | **FCT used for coding** | **Dietary intake data used to calculate the cumulative average intake** |
| --- | --- | --- |
| 1997 | FCT 1991 | 1997 |
| 2000 | FCT 1991 | Average of 1997 and 2000 |
| 2004 | FCT 2002 | Average of 1997, 2000 and 2004 |
| 2006 | FCT 2004 | Average of 1997, 2000, 2004 and 2006 |
| 2009 | FCT 2004 | Average of 1997, 2000, 2004, 2006 and 2009 |
| 2011 | FCT 2004 | Average of 1997, 2000, 2004, 2006, 2009, and 2011 |

FCT, Chinese Food Composition Table

**4. Table S2. Index to assess adherence to the TCD**

| **Component of the index** | | |  |
| --- | --- | --- | --- |
| **Food groups** | **1 point** | **2 points** |  |
| Rice |  | ≥107g but not exceeding 300g per day |  |
| Green-leafy vegetables |  | ≥257g but not exceeding 450g per day |  |
| Non-green leafy vegetables |  | ≥86g but not exceeding 150g per day |  |
| Fruits |  | ≥100g but not exceeding 150g per day |  |
| Eggs and egg products |  | ≥43g but not exceeding 100g per day |  |
| Wheat and wheat products (excluding wheat with a filling) |  | ≥71g but not exceeding 200g per day |  |
| Starchy roots and tubers |  | ≥57g but not exceeding 175g per day |  |
| Wheat with filling | ≥29g but not exceeding 150g per day |  |  |
| Pork and pork products | ≥71g but not exceeding 150g per day |  |  |
| Fermented foods | ≥21g but not exceeding 100g per day |  |  |
| Deep-fried wheat | ≥10g but not exceeding 100g per day |  |  |
| Corn and coarse grains | ≥43g but not exceeding 150g per day |  |  |
| Legume products | ≥29g but not exceeding 120g per day |  |  |
| Beef and beef products | ≥71g but not exceeding 150g per day |  |  |
| Poultry | ≥29g but not exceeding 150g per day |  |  |
| **Food-related habits** | | |  |
| Cooking by steaming and/or boiling | Yes |  |  |

TCD, traditional Chinese diet

**5. Table S3. Description of outcomes and outcome measures**

| **Outcome variable** | **Outcome measure** | **Description** | **Relative questions in the questionnaire** |
| --- | --- | --- | --- |
| Obesity | Body mass index (BMI): over 28 kg/m^2^ defined as obesity. | BMI was calculated based on measured height and weight.  Both height and weight were measured following a standard protocol recommended by the World Health Organisation and carried out by trained health workers. Weight, measured in lightweight clothing, was recorded to the nearest 0.01 kg using a calibrated beam scale. Height was measured to the nearest 0.1 cm without shoes using a portable stadiometer. | Height/weight, completed by health staff |
| Central obesity | Waist circumstance (WC): WC ≥90 cm in men and ≥80 cm in women was defined as central obesity. | WC was measured at the umbilicus level, to the nearest 0.1 cm | WC, completed by health staff |
| Hypertension | 1. Average systolic blood pressure (SBP) and diastolic blood pressure (DBP): SBP ≥ 140 mmHg or DBP ≥ 90 mmHg, or; 2. Previously diagnosed as hypertension by a doctor or taking antihypertensive drugs currently. | Mercury sphygmomanometers were used to measure SBP and DBP on the right arm in triplicate after a 10-min seated rest. The mean of the three measures was used. | 1. SBP and DBP values 2. ‘Has the doctor ever given you the diagnosis of hypertension?’ 3. ‘Have you taken any anti-hypertension drugs in the last week?’ |
| Cardiovascular diseases (CVDs) | Self-reported history. | Self-reported history of stroke and/or myocardial infarction. | 1. ‘Has the doctor ever given you a diagnosis of myocardial infarction?’ 2. ‘Has the doctor ever given you a diagnosis of stroke?’. |
| Diabetes | 1. Self-reported history or use of antidiabetic drugs, or; 2. Blood sample test results in 2009: fasting glucose levels ≥ 7.0 mmol/L (≥ 126 mg/dL), HbA1c ≥ 6.5% | Blood samples were obtained through venipuncture after an overnight fast. All samples were processed and analysed at a national central laboratory in Beijing.  Fasting serum glucose levels and routine blood tests were conducted at local hospitals using the glucose oxidase–phenol and 4-aminophenazone.  Hemoglobin A1c (HbA1c) levels were measured using high-performance liquid chromatography. | 1. ‘Has the doctor ever given you a diagnosis of diabetes?’ 2. Fasting blood glucose ≥ 7.0 mmol/L or HbA1c ≥ 6.5%) in the wave of 2009. 3. Self-reported using anti-diabetic treatment. |
| Cancer | Self-reported history. | Self-reported history of cancer. | ‘Has the doctor ever given you a diagnosis of cancer?’ |
| All-cause mortality | Household reported history | Household-reported history of death, using the earliest reported date of death when instances of several reports occurred. | ‘When did he/she move out this household? Provide date of death if the current status is death’ |

BMI, body mass index; CVD, cardiovascular disease; DBP, diastolic blood pressure; HbA1c, Hemoglobin A1c; SBP, systolic blood pressure; WC, waist circumstance

**References**

1. World Health Organisation, Collecting Step 2 data: Physical Measurements

Overview [Internet]. Available from: https://cdn.who.int/media/docs/default-source/ncds/ncd-surveillance/steps/part3-section5.pdf?sfvrsn=a46653c7_2

2. China Health and Nutrition Survey — China Health and Nutrition Survey (CHNS)

[Internet]. Available from: https://www.cpc.unc.edu/projects/china

**6. Nutrient density method for standardising TCD score**

First, the TCD score per 1000 kcal of energy intake was first calculated:

$$TCD score per 1000 kcal= \frac{Total TCD score}{Daily energy intake (kcal)/1000}$$

Second, this value was then multiplied by 2000 kcal for women and 2500 kcal for men to obtain an energy-standardised TCD score:

$$Standardised TCD score= TCD score per 1000 kcal\times2000, for women$$

$$Standardised TCD score=TCD score per 1000 kcal\times2500, for men$$

**7. Increment**

The use of increments in dietary analysis is a common method to quantify the impact of a unit change in intake on the risk of death. By standardising the intake, it allows for a more interpretable and comparable relationship between individual food group consumption and mortality. This method provides a consistent scale across different food groups, making it easier to understand the relative magnitude of effects.

Commonly, increments are defined using the standard deviation (SD) value of the food group's intake in the population, as this reflects the natural variability in consumption. For this study, each food group’s daily intake is standardised by dividing it by a specific increment value that represents a meaningful increase in intake for that group. The standardised intake for each food group was used in Cox proportional hazards regression models to estimate its effect on all-cause mortality. The standardisation formula is as follows:


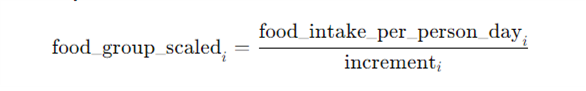


**Table S4. Increments for each food group in the Traditional Chinese Diet index**

| **Food groups** | **Increment** |  |
| --- | --- | --- |
|  |  |  |
| Rice | 186 |  |
| Wheat and wheat products (excluding wheat with fillings) | 169 |  |
| Wheat with filling | 56 |  |
| Deep-fried wheat | 59 |  |
| Corn and coarse grains | 87 |  |
| Green-leafy vegetables | 131 |  |
| Non-green leafy vegetables | 122 |  |
| Fruits | 169 |  |
| Eggs and egg products | 37 |  |
| Starchy roots and tubers | 102 |  |
| Legume products | 73 |  |
| Fermented foods | 24 |  |
| Pork and pork products | 62 |  |
| Beef and beef products | 36 |  |
| Poultry | 57 |  |

**References**

1. Zhao, J., Li, Z., Gao, Q. et al. A review of statistical methods for dietary pattern analysis. Nutr J 20, 37 (2021). <https://doi.org/10.1186/s12937-021-00692-7>
2. Naghshi S, Aune D, Beyene J, et al. Dietary intake and biomarkers of alpha linolenic acid and risk of all cause, cardiovascular, and cancer mortality: systematic review and dose-response meta-analysis of cohort studies[J]. Bmj, 2021, 375.

**8. Table S5. Intakes of 15 food groups in the TCD index**

| **Food groups** | | **Rice** | **Wheat and wheat products (excluding wheat with fillings)** | **Wheat with filling** | **Deep-fried wheat** | **Corn and coarse grains** | **Green-leafy vegetables** | **Non-green leafy vegetables** | **Fruits** | **Eggs and egg products** | **Starchy roots and tubers** | **Legume products** | **Fermented foods** | **Pork and pork products** | **Beef and beef products** | **Poultry** |
| --- | --- | --- | --- | --- | --- | --- | --- | --- | --- | --- | --- | --- | --- | --- | --- | --- |
| **Average intake (SD) (grams/day)** | | 272.5 (186.6) | 186.7 (169.7) | 56.4 (56.8) | 65.6 (59.4) | 85.4 (87.9) | 171.1 (131.8) | 130.2 (122.3) | 166.7 (169.7) | 46.1 (37.7) | 92.1 (102.2) | 83.7 (73.9) | 20.2 (24.5) | 77.6 (62.5) | 44.0 (36.0) | 69.1 (57.2) |
| **Sex** | **Average intake for men (SD) (g/day)** | 309.9 (203.9) | 205.2 (184.7) | 64.3 (68.1) | 71.8 (66.9) | 90.1 (94.3) | 178.1 (138.4) | 135.7 (129.3) | 167.8 (166.9) | 48.1 (38.5) | 95.9 (118.9) | 88.1 (78.7) | 21.5 (26.4) | 84.5 (67.2) | 46.2 (39.1) | 73.5 (62.0) |
|  | **Average intake for women (SD) (g/day** | 267.3 (165.2) | 169.6 (152.5) | 49.2 (42.8) | 59.5 (50.4) | 81.1 (81.4) | 164.6 (125.0) | 125.1 (115.2) | 165.8 (171.8) | 44.3 (36.8) | 88.6 (83.7) | 79.5 (68.9) | 18.9 (22.7) | 71.2 (57.0) | 41.6 (32.2) | 64.7 (51.5) |
|  | **p value** | <0.001 | <0.001 | 0.05 | <0.001 | <0.001 | <0.001 | <0.001 | 0.581 | <0.001 | <0.001 | <0.001 | 0.011 | <0.001 | <0.001 | <0.001 |
| **Adherence to the TCD index** | **Low adherence (0-6) (mean, SD)** | 230.2 (130.9) | 236.9 (204.8) | 68.0 (69.6) | 88.9 (95.4) | 67.2 (69.4) | 134.7 (107.0) | 107.9 (90.0) | 200.4 (239.6) | 48.4 (46.4) | 117.4 (160.0) | 92.7 (88.4) | 25.5 (29.6) | 89.3 (72.0) | 53 (46.2) | 77.5 (68.4) |
|  | **Medium adherence (7-11) (mean, SD)** | 288.4 (185.8) | 188.7 (172.4) | 61.8 (61.6) | 67.5 (56.7) | 87.1 (91.0) | 170.4 (124.8) | 124.4 (114.8) | 168.6 (182.0) | 46.0 (38.5) | 90.7 (79.8) | 82.4 (69.4) | 20.8 (24.9) | 78.9 (62.1) | 42.7 (33.0) | 68.0 (54.5) |
|  | **High adherence (12-23) (mean, SD)** | 311.4 (204.3) | 147.7 (121.6) | 37.3 (25.8) | 57.0 (41.9) | 99.7 (96.2) | 205.1 (149.5) | 161.9 (152.0) | 153.7 (125.2) | 45.5 (34.0) | 78.8 (68.0) | 77.9 (64.5) | 17.0 (20.8) | 71.0 (56.8) | 41.4 (32.8) | 65.1 (51.4) |
|  | **p value** | <0.001 | <0.001 | <0.001 | <0.001 | <0.001 | <0.001 | <0.001 | <0.001 | <0.001 | <0.001 | <0.001 | <0.001 | <0.001 | <0.001 | <0.001 |

SD, standard deviation; TCD, traditional Chinese diet; g, gram/grams.

**9. Table S6. Associations between adherence to the TCD and all-cause mortality by key demographic characteristics and lifestyle factors (sub-group analysis), CHNS, 1997-2011***

| **Subgroup** | **Adherence to the TCD** | | | |  |
| --- | --- | --- | --- | --- | --- |
|  | **Low adherence (0-6)** | **Medium adherence (7-11)** | **High adherence (12-23)** | **p _for trend_*** | **p _for interaction_*** |
| **Age group** |  |  |  |  | <0.001 |
| 18-34 | Reference | 1.09 (0.35, 3.40) | NA | 0.052 |  |
| 35-64 | Reference | 0.68 (0.51, 0.90) | 0.60 (0.47, 0.77) | <0.001 |  |
| 65-74 | Reference | 0.85 (0.64, 1.12) | 0.66 (0.51, 0.88) | <0.001 |  |
| **Sex** |  |  |  |  | 0.021 |
| Men | Reference | 0.80 (0.64, 0.98) | 0.58 (0.47, 0.73) | <0.001 |  |
| Women | Reference | 0.92 (0.72, 1.18) | 0.67 (0.51, 0.88) | 0.004 |  |
| **Household annual income** |  |  |  |  | 0.326 |
| Low | Reference | 0.71 (0.55, 0.91) | 0.45 (0.34, 0.61) | <0.001 |  |
| Medium | Reference | 0.87 (0.61, 1.26) | 0.55 (0.37, 0.81) | 0.002 |  |
| High | Reference | 1.24 (0.62, 2.47) | 1.05 (0.53, 2.08) | 0.94 |  |
| **Education level** |  |  |  |  | 0.842 |
| Low | Reference | 0.78 (0.64, 0.96) | 0.53 (0.43, 0.67) | <0.001 |  |
| Medium | Reference | 0.78 (0.34, 1.22) | 0.56 (0.24, 1.33) | 0.157 |  |
| High |  | 1.14 (0.11, 12.10) | 0.33 (0.03, 4.15) | 0.235 |  |
| **Residence area** |  |  |  |  | 0.891 |
| Rural | Reference | 0.80 (0.64, 1.00) | 0.54 (0.42, 0.69) | <0.001 |  |
| Urban | Reference | 0.72 (0.48, 1.08) | 0.50 (0.33, 0.77) | 0.002 |  |
| **Region** |  |  |  |  | 0.149 |
| North | Reference | 1.00 (0.69, 1.45) | 0.54 (0.37, 0.79) | <0.001 |  |
| South | Reference | 0.70 (0.55, 0.88) | 0.53 (0.40, 0.69) | <0.001 |  |
| **PA level** |  |  |  |  | 0.965 |
| No activity | Reference | 0.68 (0.22, 2.09) | 0.29 (0.08, 1.09) | 0.055 |  |
| Very light | Reference | 0.64 (0.44, 0.93) | 0.47 (0.33, 0.69) | <0.001 |  |
| Light | Reference | 0.86 (0.57, 1.31) | 0.62 (0.40, 0.95) | 0.025 |  |
| Moderate | Reference | 0.99 (0.49, 2.00) | 0.63 (0.28, 1.41) | 0.268 |  |
| Heavy | Reference | 0.81 (0.59, 1.12) | 0.51 (0.34, 0.75) | <0.001 |  |
| Very heavy | Reference | NA | NA |  |  |
| **Smoking status** |  |  |  |  | 0.121 |
| Non-smoker | Reference | 0.65 (0.50,0.85) | 0.45 (0.34,0.59) | <0.001 |  |
| Ex-smoker | Reference | 2.64 (1.05, 6.63) | 1.13 (0.44, 2.92) | 0.978 |  |
| Current smoker | Reference | 0.92 (0.67, 1.25) | 0.61 (0.43, 0.87) | 0.006 |  |

CHNS, Chinese Health Nutrition Survey; TCD, traditional Chinese diet.

***** Analyses were conducted using Cox proportional hazards models. Interaction effects between TCD adherence and each subgroup variable were assessed using the Wald test. The p-values were adjusted using the Bonferroni correction; p < 0.002 considered statistically significant.

Models were adjusted for age, sex, residence area (urban/rural), education level, household annual income, alcohol consumption (yes/no), smoking status, PA level, BMI, anti-hypertension drugs and diabetes treatments. Stratification variables were not adjusted in the corresponding models.

**10.** **Sensitivity analyses**

**10.1 Sensitivity analyses 1**

**Table S7a. Associations between TCD adherence and NCD-related outcomes, using nutrient density method, CHNS, 1997-2011***

| **NCD-related outcomes** | **Adherence to the TCD** | | | | |  |
| --- | --- | --- | --- | --- | --- | --- |
|  | **Low adherence^a^** | **Medium adherence^a^** |  | **High adherence^a^** |  |  |
|  | OR (95%CI) | OR (95%CI) | p value | OR (95%CI) | p value** | p for trend*** |
| **CVD** |  |  |  |  |  |  |
| Model 1 | Reference | 0.67 (0.58, 0.78) | <0.001 | 0.54 (0.46, 0.64) | <0.001 | <0.001 |
| Model 2 | Reference | 0.79 (0.68, 0.93) | 0.004 | 0.77 (0.63, 0.94) | 0.009 | 0.004 |
| Model 3 | Reference | 0.81 (0.69, 0.95) | 0.009 | 0.80 (0.66, 0.97) | 0.034 | 0.016 |
| **Diabetes** |  |  |  |  |  |  |
| Model 1 | Reference | 1.02 (0.84, 1.26) | 0.870 | 0.68 (0.52, 0.88) | 0.004 | 0.010 |
| Model 2 | Reference | 1.18 (0.94, 1.49) | 0.158 | 0.86 (0.63, 1.17) | 0.323 | 0.585 |
| Model 3 | Reference | 1.32 (0.99, 1.74) | 0.054 | 0.88 (0.61, 1.28) | 0.510 | 0.819 |
| **Cancer** |  |  |  |  |  |  |
| Model 1 | Reference | 1.02 (0.64, 1.63) | 0.919 | 0.41 (0.19, 0.85) | 0.017 | 0.040 |
| Model 2 | Reference | 1.34 (0.81, 2.22) | 0.250 | 0.70 (0.31, 1.58) | 0.391 | 0.774 |
| Model 3 | Reference | 1.36 (0.82, 2.24) | 0.234 | 0.70 (0.31, 1.57) | 0.395 | 0.788 |
| **Obesity** |  |  |  |  |  |  |
| Model 1 | Reference | 1.03 (0.94, 1.13) | 0.480 | 0.99 (0.91, 1.10) | 0.964 | 0.931 |
| Model 2 | Reference | 0.95 (0.87, 104) | 0.324 | 0.86 (0.78, 0.95) | 0.003 | 0.003 |
| Model 3 | Reference | NA | NA | NA | NA | NA |
| **Central obesity** |  |  |  |  |  |  |
| Model 1 | Reference | 1.00 (0.95, 1.06) | 0.860 | 1.03 (0.98, 1.09) | 0.272 | 0.267 |
| Model 2 | Reference | 0.89 (0.85, 0.95) | <0.001 | 0.85 (0.80, 0.91) | <0.001 | <0.001 |
| Model 3 | Reference | NA | NA | NA | NA | NA |
| **Hypertension** |  |  |  |  |  |  |
| Model 1 | Reference | 0.82 (0.75, 0.89) | <0.001 | 0.64 (0.58, 0.71) | <0.001 | <0.001 |
| Model 2 | Reference | 0.94 (0.86, 1.03) | 0.204 | 0.85 (0.76, 0.95) | 0.005 | 0.006 |
| Model 3 | Reference | 0.94 (0.80, 1.10) | 0.437 | 0.90 (0.74, 1.08) | 0.251 | 0.246 |

CHNS, Chinese Health Nutrition Survey; CIs, confidence intervals; NCD, non-communicable disease; OR, odds ratio; TCD, traditional Chinese diet; NA, not applicable.

*****  All analyses were conducted using multilevel logistic regression models incorporating time variations and random effects.

** The p-value was adjusted (p<0.008) for multiple comparisons using the Bonferroni correction.

*** This p-value was used to assess the linear trend

^a^ Low, medium, and high adherence were defined based on tertiles of the energy-standardised TCD score

Model 1: Crude model

Model 2: Adjusted for age, sex, residence area (urban/rural), education level, household annual income, alcohol consumption (yes/no), smoking status, PA level and energy intake.

Model 3: Adjusted for variables in Model 2, in addition to BMI, anti-hypertension medication and diabetes treatment

All adjusted variables treated as time-varying covariates.

**{Popkin, 2006 #2905}Table S7b. Associations between TCD adherence and all-cause mortality, using nutrient density method, CHNS, 1997-2011***

| **Variable** |  | **Adherence to the TCD**** | | | | |  |
| --- | --- | --- | --- | --- | --- | --- | --- |
|  | **Low adherence^a^** | **Medium adherence^a^** | **p value** | **High adherence^a^** | **p value** | **Per SD TCD score increment** | **p value** |
| **Person-year of follow-up** | 19,969 | 33,444 |  | 42,519 |  | 95,932 |  |
| **Events, No.** | 152 | 198 |  | 236 |  | 586 |  |
| **Model 1 (HR, 95%CI)** | Reference | 0.80 (0.68,0.94) | 0.006 | 0.61 (0.53,0.70) | <0.001 | 0.75 (0.67, 0.84) | 0.002 |
| **Model 2 (HR, 95%CI)** | Reference | 0.83 (0.69, 0.92) | 0.017 | 0.66 (0.55,0.72) | <0.001 | 0.85 (0.77, 0.95) | <0.001 |
| **Model 3 (HR, 95%CI)** | Reference | 0.84 (0.71, 0.92) | 0.010 | 0.67 (0.54,0.74) | <0.001 | 0.86 (0.76, 0.95) | 0.008 |

CHNS, Chinese Health Nutrition Survey; CIs, confidence intervals; HR, hazard ratio; SD, standard deviation; TCD, traditional Chinese diet.

* Analyses were conducted using Cox proportional hazards models.

^a^ Low, Medium, and High adherence were defined based on tertiles of the energy- standardised TCD score

Model 1: Crude model

Model 2: Adjusted for age, sex, residence area (urban/rural), education level, household annual income, alcohol consumption (yes/no), smoking status, PA level, and energy intake.

Model 3: Adjusted for variables in Model 2, in addition to BMI, anti-hypertension medication and diabetes treatment

All adjusted variables were treated as time-varying covariates.

**10.2 Sensitivity analyses 2**

**Table S7c. Associations between TCD adherence and NCD-related outcomes, using the equal scores classification method, CHNS, 1997-2011***

| **NCD-related outcomes** | **Adherence to the TCD** | | | | |  |
| --- | --- | --- | --- | --- | --- | --- |
|  | **Low adherence (0-7)** | **Medium adherence (8-15)** |  | **High adherence (16-23)** |  |  |
|  | OR (95%CI) | OR (95%CI) | p value** | OR (95%CI) | p value** | p for trend*** |
| **CVD** |  |  |  |  |  |  |
| Model 1 | Reference | 0.77 (0.65, 0.91) | 0.002 | 0.55 (0.45, 0.66) | <0.001 | 0.001 |
| Model 2 | Reference | 0.90 (0.76, 1.07) | 0.221 | 0.73 (0.59, 0.89) | 0.002 | 0.001 |
| Model 3 | Reference | 0.94 (0.74, 1.06) | 0.175 | 0.76 (0.62, 0.94) | 0.009 | 0.008 |
| **Diabetes** |  |  |  |  |  |  |
| Model 1 | Reference | 1.06 (0.84, 1.36) | 0.599 | 0.73 (0.55, 0.97) | 0.033 | 0.023 |
| Model 2 | Reference | 1.20 (0.93, 1.54) | 0.152 | 0.88 (0.65. 1.19) | 0.404 | 0.362 |
| Model 3 | Reference | 1.25 (0.91, 1.72) | 0.161 | 0.97 (0.67, 1.40) | 0.861 | 0.717 |
| **Cancer** |  |  |  |  |  |  |
| Model 1 | Reference | 1.20 (0.69, 2.10) | 0.515 | 0.84 (0.42, 1.67) | 0.611 | 0.587 |
| Model 2 | Reference | 1.28 (0.73, 2.25) | 0.388 | 0.97 (0.48, 1.96) | 0.931 | 0.931 |
| Model 3 | Reference | 1.27 (0.73, 2.24) | 0.399 | 0.97 (0.48, 1.95) | 0.925 | 0.924 |
| **Obesity** |  |  |  |  |  |  |
| Model 1 | Reference | 1.22 (1.12, 1.33) | <0.001 | 1.44 (1.28, 1.61) | <0.001 | <0.001 |
| Model 2 | Reference | 1.15 (1.06, 1.26) | 0.001 | 1.27 (1.13, 1.43) | <0.001 | <0.001 |
| Model 3 | Reference | NA | NA | NA | NA | NA |
| **Central obesity** |  |  |  |  |  |  |
| Model 1 | Reference | 1.24 (1.19, 1.30) | <0.001 | 1.46 (1.36, 1.56) | <0.001 | <0.001 |
| Model 2 | Reference | 1.13 (1.08, 1.19) | <0.001 | 1.21 (1.13, 1.31) | <0.001 | <0.001 |
| Model 3 | Reference | NA | NA | NA | NA | NA |
| **Hypertension** |  |  |  |  |  |  |
| Model 1 | Reference | 0.89 (0.81, 0.98) | 0.017 | 0.69(0.61, 0.76) | <0.001 | <0.001 |
| Model 2 | Reference | 1.01 (0.92, 1.12) | 0.784 | 0.86 (0.76, 0.96) | 0.010 | 0.002 |
| Model 3 | Reference | 0.98 (0.79, 1.20) | 0.829 | 0.84 (0.67, 1.06) | 0.137 | 0.079 |

CHNS, Chinese Health Nutrition Survey; CIs, confidence intervals; NCD, non-communicable disease; OR, odds ratio; TCD, traditional Chinese diet; NA, not applicable.

* All analyses were conducted using multilevel logistic regression models incorporating time variations and random effects.

** The p-value was adjusted (p<0.008) for multiple comparisons using the Bonferroni correction; p<0.008 are considered statistically significant.

*** This p-value was used to assess the linear trend

Model 1: Crude model

Model 2: Adjusted for age, sex, residence area (urban/rural), education level, household annual income, alcohol consumption (yes/no), smoking status, PA level and energy intake.

Model 3: Adjusted for variables in Model 2, in addition to BMI, anti-hypertension medication and diabetes treatment

All adjusted variables were treated as time-varying covariates.

**Table S7d. Associations between TCD adherence and all-cause mortality, using the equal scores classification method, CHNS, 1997-2011***

|  | **Adherence to the TCD** | | | |  |  |  |
| --- | --- | --- | --- | --- | --- | --- | --- |
| **Variable** | **Low adherence (0-7)** | **Medium adherence (8-15)** | **p value** | **High adherence (16-23)** | **p value** | **Per SD TCD score increment** | **p value** |
| **Person-year of follow-up** | 25,361 | 49,703 |  | 20,541 |  | 95,605 |  |
| **Events, No.** | 243 | 273 |  | 70 |  | 586 |  |
| Model 1 | Reference | 0.56 (0.47, 0.66) | <0.001 | 0.35 (0.27, 0.46) | <0.001 | 0.58 (0.52, 0.66) | <0.001 |
| Model 2 | Reference | 0.66 (0.55, 0.78) | <0.001 | 0.50 (0.38, 0.66) | <0.001 | 0.80 (0.70, 0.88) | <0.001 |
| Model 3 | Reference | 0.68 (0.56, 0.80) | <0.001 | 0.51 (0.38, 0.66) | <0.001 | 0.80 (0.71, 0.90) | <0.001 |

CHNS, Chinese Health Nutrition Survey; CIs, confidence intervals; SD, standard deviation; TCD, traditional Chinese diet.

* Analyses were conducted using Cox proportional hazards models. The p-values were adjusted using the Bonferroni correction; p < 0.008 considered statistically significant.

Model 1: Crude model

Model 2: Adjusted for age, sex, residence area (urban/rural), education level, household annual income, alcohol consumption (yes/no), smoking status, PA level and energy intake.

Model 3: Adjusted for variables in Model 2, in addition to BMI, anti-hypertension medication and diabetes treatment

All adjusted variables were treated as time-varying covariates.

**10.3 Sensitivity analyses 3**

**Table S7e. Associations between TCD adherence and NCD-related outcomes, using the three-day mean dietary intake, CHNS, 1997-2011***

| **NCD-related outcomes** | **Adherence to the TCD** | | | | |  |
| --- | --- | --- | --- | --- | --- | --- |
|  | **Low adherence (0-4)** | **Medium adherence (5-8)** |  | **High adherence (9-23)** |  |  |
|  | OR (95%CI) | OR (95%CI) | p value** | OR (95%CI) | p value** | p for trend*** |
| **CVD** |  |  |  |  |  |  |
| Model 1 | Reference | 0.86 (0.74, 1.00) | 0.056 | 0.74 (0.62, 0.88) | <0.001 | <0.001 |
| Model 2 | Reference | 0.90 (0.73, 1.04) | 0.155 | 0.78 (0.66, 0.94) | 0.007 | 0.007 |
| Model 3 | Reference | 0.91 (0.77, 1.06) | 0.224 | 0.81 (0.68, 0.97) | 0.022 | 0.022 |
| **Diabetes** |  |  |  |  |  |  |
| Model 1 | Reference | 0.93 (0.77, 1.14) | 0.505 | 0.71 (0.59, 0.86) | <0.001 | <0.001 |
| Model 2 | Reference | 0.98 (0.82, 1.18) | 0.851 | 0.80 (0.67, 0.96) | 0.014 | 0.007 |
| Model 3 | Reference | 1.23 (0.88, 1.73) | 0.225 | 1.09 (0.80, 1.50) | 0.581 | 0.733 |
| **Cancer** |  |  |  |  |  |  |
| Model 1 | Reference | 0.97 (0.59, 1.58) | 0.897 | 0.68 (0.37, 1.27) | 0.224 | 0.262 |
| Model 2 | Reference | 1.01 (0.62, 1.66) | 0.956 | 0.72 (0.39, 1.34) | 0.295 | 0.187 |
| Model 3 | Reference | 1.01 (0.62, 1.66) | 0.968 | 0.72 (0.38, 1.33) | 0.294 | 0.187 |
| **Obesity** |  |  |  |  |  |  |
| Model 1 | Reference | 1.11 (1.01, 1.22) | 0.023 | 1.16 (1.05, 1.28) | 0.003 | 0.003 |
| Model 2 | Reference | 1.07 (0.98, 1.18) | 0.130 | 1.08 (0.98. 1.20) | 0.130 | 0.128 |
| Model 3 | Reference | NA | NA | NA | NA | NA |
| **Central obesity** |  |  |  |  |  |  |
| Model 1 | Reference | 1.12 (1.07, 1.18) | <0.001 | 1.14 (1.08, 1.21) | <0.001 | <0.001 |
| Model 2 | Reference | 1.09 (1.03, 1.15) | 0.002 | 1.07 (1.01, 1.14) | 0.027 | 0.019 |
| Model 3 | Reference | NA | NA | NA | NA |  |
| **Hypertension** |  |  |  |  |  |  |
| Model 1 | Reference | 0.91 (0.83. 0.99) | 0.022 | 0.83 (0.75, 0.91) | <0.001 | <0.001 |
| Model 2 | Reference | 0.94 (0.86, 1.03) | 0.200 | 0.88 (0.80, 0.97) | 0.013 | 0.013 |
| Model 3 | Reference | 0.94 (0.79, 1.12) | 0.508 | 0.83 (0.67, 1.00) | 0.051 | 0.050 |

CHNS, Chinese Health Nutrition Survey; CIs, confidence intervals; NCD, non-communicable disease; OR, odds ratio; TCD, traditional Chinese diet; NA, not applicable.

* All analyses were conducted using multilevel logistic regression models incorporating time variations and random effects.

** The p-value was adjusted (p<0.008) for multiple comparisons using the Bonferroni correction; p<0.008 are considered statistically significant.

*** This p-value was used to assess the linear trend

Model 1: Crude model

Model 2: Adjusted for age, sex, residence area (urban/rural), education level, household annual income, alcohol consumption (yes/no), smoking status, PA level and energy intake.

Model 3: Adjusted for variables in Model 2, in addition to BMI, anti-hypertension medication and diabetes treatment

All adjusted variables were treated as time-varying covariates.

**Table S7f. Associations between TCD adherence and all-cause mortality, using the three-day mean dietary intake, CHNS, 1997-2011***

| **Variable** | **Low adherence (0-4)** | **Medium adherence (5-8)** | **p value**** | **High adherence (9-23)** | **p value**** | **Per SD TCD score increment** | | **p value**** |
| --- | --- | --- | --- | --- | --- | --- | --- | --- |
| **Person-year of follow-up** | 22,248 | 30,908 |  | 43,093 |  | | 96,249 |  |
| **Events, No.** | 216 | 204 |  | 167 |  | | 587 |  |
| **Model 1** | Reference | 0.76 (0.65, 0.90) | <0.001 | 0.68 (0.61, 0.76) | <0.001 | | 0.82 (0.75, 0.88) | <0.001 |
| **Model 2** | Reference | 0.86 (0.74, 0.91) | <0.001 | 0.75 (0.67, 0.87) | <0.001 | | 0.91 (0.86, 0.95) | <0.001 |
| **Model 3** | Reference | 0.86 (0.74, 0.92) | <0.001 | 0.76 (0.66, 0.85) | <0.001 | | 0.91 (0.85, 0.97) | 0.002 |

CHNS, Chinese Health Nutrition Survey; CIs, confidence intervals; SD, standard deviation; TCD, traditional Chinese diet.

* Analyses were conducted using Cox proportional hazards models. The p-values were adjusted using the Bonferroni correction; p < 0.008 considered statistically significant.

Model 1: Crude model

Model 2: Adjusted for age, sex, residence area (urban/rural), education level, household annual income, alcohol consumption (yes/no), smoking status, PA level and energy intake.

Model 3: Adjusted for variables in Model 2, in addition to BMI, anti-hypertension medication and diabetes treatment

All adjusted variables were treated as time-varying covariates.

**11.** **Baseline subgroup participant characteristics**

**Table S8. Baseline participant characteristics by socioeconomic factors***

| **Characteristic** | **Annual household income (CNY/year)^a^** | | | | **Education level** | | | | **Residence area** | | |
| --- | --- | --- | --- | --- | --- | --- | --- | --- | --- | --- | --- |
|  | **Low** | **Medium** | **High** | **p value** | **Low** | **Medium** | **High** | **p value** | **Rural** | **Urban** | **p value** |
| **N** | 6,816 | 3,394 | 948 |  | 8,713 | 2,061 | 384 |  | 7,315 | 3,843 |  |
| **Age, years, mean (SD)** | 42.5 (13.4) | 41.9 (13.3) | 40.9 (14.0) | 0.001 | 43.1 (13.5) | 38.7 (12.5) | 39.6 (13.7) | <0.001 | 41.7 (13.1) | 43.0 (13.9) | <0.001 |
| **Sex** |  |  |  | 0.435 |  |  |  | <0.001 |  |  | 0.163 |
| **Male, n (%)** | 3,330 (48.9) | 1,626 (47.9) | 475 (50.1) |  | 4,026 (46.2) | 1,172 (56.9) | 233 (60.7) |  | 3,596 (49.2) | 1,835 (47.7) |  |
| **Female, n (%)** | 3,486 (51.1) | 1,768 (52.1) | 473 (49.9) |  | 4,687 (53.8) | 889 (43.1) | 151 (39.3) |  | 3,719 (50.8) | 2,008 (52.3) |  |
| **BMI, kg/m², mean (SD)** | 22.5 (3.1) | 23.0 (3.3) | 23.2 (3.4) | <0.001 | 22.7 (3.2) | 22.9 (3.1) | 22.7 (3.1) | 0.040 | 22.6 (3.2) | 23.0 (3.3) | <0.001 |
| **TCD score (0–23), mean (SD)** | 6.56 (4.18) | 7.77 (4.89) | 9.68 (5.37) | <0.001 | 6.92 (4.44) | 8.01 (5.02) | 9.12 (5.21) | <0.001 | 6.87 (4.35) | 7.80 (5.03) | <0.001 |
| **TCD adherence** |  |  |  | <0.001 |  |  |  | <0.001 |  |  | <0.001 |
| **Low (0–6), n (%)** | 3,738 (54.8) | 1,523 (44.9) | 280 (29.5) |  | 4,538 (52.1) | 878 (42.6) | 125 (32.6) |  | 3,825 (52.3) | 1,716 (44.7) |  |
| **Medium (7–11), n (%)** | 2,212 (32.5) | 1,167 (34.4) | 338 (35.7) |  | 2,869 (32.9) | 703 (34.1) | 145 (37.8) |  | 2,426 (33.2) | 1,291 (33.6) |  |
| **High (12–23), n (%)** | 866 (12.7) | 704 (20.7) | 330 (34.8) |  | 1,306 (15.0) | 480 (23.3) | 114 (29.7) |  | 1,064 (14.5) | 836 (21.8) |  |
| **Smoking status** |  |  |  | <0.001 |  |  |  | 0.030 |  |  | <0.001 |
| **Non-smoker, n (%)** | 4,580 (67.2) | 2,312 (68.1) | 676 (71.3) |  | 5,899 (67.7) | 1,393 (67.6) | 276 (71.9) |  | 4,908 (67.1) | 2,660 (69.2) |  |
| **Ex-smoker, n (%)** | 40 (0.6) | 45 (1.3) | 18 (1.9) |  | 74 (0.8) | 21 (1.0) | 8 (2.1) |  | 52 (0.7) | 51 (1.3) |  |
| **Current smoker, n (%)** | 2,196 (32.2) | 1,037 (30.6) | 254 (26.8) |  | 2,740 (31.4) | 647 (31.4) | 100 (26.0) |  | 2,355 (32.2) | 1,132 (29.5) |  |
| **PA level** |  |  |  | <0.001 |  |  |  | <0.001 |  |  | <0.001 |
| **No activity, n (%)** | 60 (0.9) | 35 (1.0) | 4 (0.4) |  | 84 (1.0) | 9 (0.4) | 6 (1.6) |  | 42 (0.6) | 57 (1.5) |  |
| **Very light, n (%)** | 997 (14.6) | 716 (21.1) | 243 (25.6) |  | 1,119 (12.8) | 645 (31.3) | 192 (50.0) |  | 804 (11.0) | 1,152 (30.0) |  |
| **Light, n (%)** | 1,790 (26.3) | 1,038 (30.6) | 342 (36.1) |  | 2,320 (26.6) | 716 (34.7) | 134 (34.9) |  | 1,880 (25.7) | 1,290 (33.6) |  |
| **Moderate, n (%)** | 900 (13.2) | 575 (16.9) | 180 (19.0) |  | 1,195 (13.7) | 411 (19.9) | 49 (12.8) |  | 955 (13.1) | 700 (18.2) |  |
| **Heavy, n (%)** | 3,020 (44.3) | 1,020 (30.1) | 171 (18.0) |  | 3,931 (45.1) | 277 (13.4) | 3 (0.8) |  | 3,580 (48.9) | 631 (16.4) |  |
| **Very heavy, n (%)** | 49 (0.7) | 10 (0.3) | 8 (0.8) |  | 64 (0.7) | 3 (0.1) | 0 (0.0) |  | 54 (0.7) | 13 (0.3) |  |

BMI, body mass index; PA, physical activity; SD, standard deviation; TCD, traditional Chinese diet

* Participants included at baseline (n = 11,158). Continuous variables are reported as mean (SD) and categorical variables as n (%). Significance was assessed using chi-square test for categorical variables or ANOVA/Kruskal–Wallis for continuous variables.

^a^ CNY/Y = Chinese Yuan per year. Household income was calculated as the total income and revenue from all sources, subtracting expenditures, and then adjusted for inflation to reflect 2015 values in CNY/Yuan.

**12. Table S9. Concordance between self-reported and biomarker-defined diabetes in the 2009 wave sub-sample, CHNS (n=5,678) ***

|  | **Estimate (95% CI)** |
| --- | --- |
| **Overall agreement, %** | 91.3 (90.5–92.0) |
| **Cohen's κ** | 0.308 (0.250–0.367) |
| **Sensitivity, %** | 22.2 (19.0–25.8) |
| **Specificity, %** | 99.1 (98.8–99.3) |
| **Positive predictive value, %** | 73.6 (66.6–79.6) |
| **Negative predictive value, %** | 91.8 (91.1–92.5) |

CHNS, China Health and Nutrition Survey; CI, confidence interval
